# Indirubin Derivative 7-Bromoindirubin-3-Oxime (7Bio) Attenuates Aβ Oligomer-Induced Cognitive Impairments in Mice

**DOI:** 10.3389/fnmol.2017.00393

**Published:** 2017-11-28

**Authors:** Liping Chen, Chunhui Huang, Jieyi Shentu, Minjun Wang, Sicheng Yan, Fei Zhou, Zaijun Zhang, Chuang Wang, Yifan Han, Qinwen Wang, Wei Cui

**Affiliations:** ^1^Ningbo Key Laboratory of Behavioral Neuroscience, Zhejiang Provincial Key Laboratory of Pathophysiology, School of Medicine, Ningbo University, Ningbo, China; ^2^Laboratory of Marine Natural Products, School of Marine Sciences, Ningbo University, Ningbo, China; ^3^Institute of New Drug Research, Guangdong Province Key Laboratory of Pharmacodynamic, Constituents of Traditional Chinese Medicine and New Drug Research, College of Pharmacy, Jinan University, Guangdong, China; ^4^Department of Applied Biology and Chemistry Technology, Institute of Modern Chinese Medicine, Hong Kong Polytechnic University, Hong Kong, China

**Keywords:** Alzheimer's disease, 7-bromoindirubin-3-oxime, β-amyloid, CDK5, GSK3β

## Abstract

Indirubins are natural occurring alkaloids extracted from indigo dye-containing plants. Indirubins could inhibit various kinases, and might be used to treat chronic myelocytic leukemia, cancer and neurodegenerative disorders. 7-bromoindirubin-3-oxime (7Bio), an indirubin derivative derived from indirubin-3-oxime, possesses inhibitory effects against cyclin-dependent kinase-5 (CDK5) and glycogen synthase kinase-3β (GSK3β), two pharmacological targets of Alzheimer's disease (AD). In this study, we have discovered that 2.3–23.3 μg/kg 7Bio effectively prevented β-amyloid (Aβ) oligomer-induced impairments of spatial cognition and recognition without affecting bodyweight and motor functions in mice. Moreover, 7Bio potently inhibited Aβ oligomer-induced expression of interleukin-6 (IL-6) and tumor necrosis factor-α (TNF-α). Furthermore, 7Bio significantly prevented the decreased expression of synapsin-1 and PSD-95, biomarkers of pre-synaptic and post-synaptic proteins in Aβ oligomer-treated mice. The mean optical density (OD) with hyper-phosphorylated tau (pTau), glial fibrillary acidic protein (GFAP) and CD45 positive staining in the hippocampus of 7Bio-treated mice were significantly decreased compared to those of Aβ oligomer-treated mice. In addition, Western blotting analysis showed that 7Bio attenuated Aβ oligomer-decreased expression of pSer9-GSK3β. Those results suggested that 7Bio could potently inhibit Aβ oligomer-induced neuroinflammation, synaptic impairments, tau hyper-phosphorylation, and activation of astrocytes and microglia, which may contribute to the neuroprotective effects of 7Bio. Based on these findings, we expected that 7Bio might be developed as a novel anti-AD lead compound.

## Introduction

Indirubins are indoline alkaloids which were extracted from indigo dye-containing natural plants (Gaboriaud-Kolar et al., 2015; Fang et al., 2017). Moreover, indirubins could be detected in human urine, suggesting that these compounds could be produced by human body (Fang et al., 2017). Previous studies have shown that indirubins could inhibit various kinases, including cyclin-dependent kinases (CDKs) and glycogen synthase kinase-3β (GSK3β) in particular, via competitively occupying their ATP binding sites (Ribas et al., 2006; Begum et al., 2015). Therefore, indirubins could be used to treat various diseases related to the abnormity of kinases, such as cancer and chronic myelocytic leukemia. However, the poor bioavailability of indirubins drives the development and synthesis of new indirubin derivatives with improved druggability and anti-cancer activity (Eisenbrand et al., 2004; Kurita et al., 2016).

7-bromoindirubin-3-oxime (7Bio), a novel indirubin derivative with a bromine molecule in the aromatic ring of indirubin-3-oxime, was one of the most potent indirubin derivatives to induce the death of cancer cells (Ribas et al., 2006). 7Bio at 25 μM could lead to apoptosis in many tumor cell lines, such as SH-SY5Y cells, Jurkat cells, and MDA-MB-231 cells (Nicolaou et al., 2012). 7Bio also possesses inhibitory effects against many kinases. It was revealed that 7Bio inhibits the activities of CDK1, CDK5, and GSK3β with IC_50_s at 22, 33 and 32 μM, respectively (Ribas et al., 2006).

CDKs and GSK3β are also targets for many neurodegenerative disorders, such as Alzheimer's disease (AD) and Parkinson's disease (PD). Many neurotoxins, including 1-methyl-4-phenylpyridinium ion and β-amyloid (Aβ), could enhance the activities of CDK5 and GSK3β, inducing neuroinflammation, neuronal loss, astrogliosis and activation of microglia, eventually leading to the impairments of cognition and motor functions (Mushtaq et al., 2016). Many studies have demonstrated that indirubins could produce neuroprotective effects both *in vitro* and *in vivo* (Zhang et al., 2009; Hu et al., 2015). Indirubin-3-oxime and indirubin could prevent 6-hydroxydopamine, H_2_O_2_, Aβ and potassium deprivation-induced neuronal apoptosis *in vitro* (Hu et al., 2015; Yu et al., 2017b). Moreover, indirubin-3-oxime protected against behavioral abnormities induced by 1-methyl-4-phenyl-1,2,3,6-tetrahydropyridine in rodents (Wang et al., 2007; Ding et al., 2010). Furthermore, indirubin-3-oxime rescued spatial memory deficits in transgenic APP/PS1 mice (Ribas et al., 2006; Ding et al., 2010). However, the detailed molecular mechanisms underlying the neuroprotective effects of indirubin derivatives are largely unknown.

In this study, we have evaluated the effects of 7Bio on cognitive impairments in Aβ oligomer-treated mice. We found that 2.3–23.3 μg/kg 7Bio effectively prevented Aβ oligomer-induced impairments of spatial cognition and recognition without affecting bodyweight and motor functions. Moreover, 7Bio potently inhibited Aβ oligomer-induced neuroinflammation, synaptic damage, tau hyper-phosphorylation, astrogliosis and activation of microglia, possibly via inhibiting GSK3β, which might concurrently contribute to the neuroprotective effects of 7Bio.

## Materials and methods

### Chemicals and reagents

7-Bromoindirubin-3′-oxime, indirubin-3-oxime, indirubin-3-(2,3-dihydroxypropyl) oximether, indirubin-3- monoxime-5-sulphonic acid, 5-iodo-indirubin-3-monoxime, indirubin derivative E804 were was purchased from Santa Cruz Biotechnology (Dallas, TX, USA). Synthetic Aβ_1–42_ peptide was obtained from GL Biochem (Shanghai, China).

### SH-SY5Y cells culture

SH-SY5Y cells were maintained in high glucose modified Eagle's medium (DMEM) supplemented with 10% fetal bovine serum and penicillin (100 U/ml)/streptomycin (100 μg/ml) at 37°C with 5% CO_2_. The medium was refreshed every two days. Before experiments, SH-SY5Y cells were seeded in DMEM with 1% fetal bovine serum for 24 h.

### Cell viability measurements

Cell viability was measured by 3(4,5-dimethylthiazol-2-yl)-2.5-diphenyltetrazolium bromide (MTT) assay. Briefly, 10 μl MTT (5 mg/ml) was added to each well in 96-well plates. Then, plates were incubated at 37°C for 4 h, and 100 μl solvate (0.01 N HCl in 10% SDS) was added. After 16–20 h, the absorbance of samples was measured at a wavelength of 570 nm with 655 nm as a reference wavelength.

### Dot blotting analysis

The nitrocellulose membrane was divided into equal grids. Subsequently, a 2 μL sample was spotted onto the membrane and then air-dried. The membrane was blocked in a TBST (50 mM Tris, 150 mM NaCl, and 0.1% Tween-20) solution containing 10% milk overnight and then incubated with anti-oligomer antibody A11 (Thermo Fisher Scientific, Waltham, MA, USA, 1:1,000) or anti-Aβ1-17 antibody 6E10 (Sigma, 1:1,000) for 1 h with gentle shaking. After three washes with TBST, the membrane was incubated with secondary antibodies for 1 h and developed with an enhanced chemiluminescence plus kit.

### Preparation of Aβ_1−42_ oligomer

Soluble Aβ_1−42_ oligomer was obtained as previously described (Xiang et al., 2017). Briefly, Aβ_1−42_ was added in hexafluoroisopropanol (HFIP, Sigma, St. Louis, MO, USA) to become Aβ_1−42_ monomers. Aβ_1−42_ monomers were spin-vacuumed in 10% HFIP solution. Then, HFIP was evaporated to obtain Aβ_1−42_ solution. Aβ_1−42_ solution was incubated for 2 days at 25°C under stirring, and centrifuged at 14,000 g for 15 min at 4°C. The supernatant contains mainly soluble Aβ_1−42_ oligomer. The supernatant was collected and quantified by BCA assay (Thermo Fisher Scientific, Waltham, MA, USA).

### Drug treatment for animal study

Use and care of mice followed the guidelines of Ningbo University Animal Research Advisory Committee. Male Institute of Cancer Research (ICR) mice weighing around 30 g (8 weeks age) were provided by Zhejiang Academy of medical Sciences (Hangzhou, Zhejiang, China). Animals were kept with a 12-h light/dark cycle (humidity: 50 ± 10%) at 22 ± 2°C, and standard food and water were provided.

7Bio were dissolved in MilliQ water. Mice were randomly assigned into five groups with ten animals in each group as follows: control, Aβ_1−42_ oligomer, Aβ_1−42_ oligomer plus low (2.3 μg/kg), medium (7.0 μg/kg) and high (23.3 μg/kg) concentrations of 7Bio. Mice were anesthetized by intraperitoneal (i.p.) administration of sodium pentobarbital (50 mg/kg) before placed in a stereotaxic apparatus (RWD life science, Shenzhen, China). Cannulae (30-gauge, 6 mm; RWD life science) were implanted into bilateral ventricle regions using the following coordinate: AP −0.4 mm from bregma; ML ±1.0 mm from the midline; and DV −2.0 mm from pia mater. The cannulae were anchored to the skull with dental cement. The mice were allowed to recover for 6 days. On day 7, Aβ_1−42_ oligomer were administrated into bilateral ventricle regions for 3days, 7Bio with Aβ_1−42_ oligomer or saline were administrated into bilateral ventricle regions for 5 days. Microinfusions (1 μl/side) of drugs were injected for over 5 min. The cannulas were left in place for another 5 min to minimize backflow. For mice in the control group, cannulas were implanted to inject the vehicle in the ventricle regions. Many studies have shown that injection of Aβ oligomer in the ventricle regions of ICR mice could produce cognitive impairments (Kim et al., 2016; Xiang et al., 2017).

### Open field test

To analyze the exploratory behavior and locomotor activity, animals were placed in the left rear quadrant of an open field (50 × 50 × 39 cm) with white plywood walls and a brown floor divided into four equal squares of equal dimensions (25 × 25 cm) (Wu et al., 2017). The animals were placed one by one at the center of the box and allowed to explore it for 5 min. Hand-operated counters and stopwatches were used to score the number of line crossing with four paws and the number of rearing (number of times the animals stood on its hind legs), which were used as indicators of exploratory behavior and locomotor activity, respectively. The drug status of the subjects was blindly monitored by an independent investigator. To avoid perturbation of the animals due to urine and feces, the open field was cleaned with 10% ethanol solution and dry cloth between two tests.

### Novel object recognition (NOR) tests

The NOR tests were conducted in an open-field arena (30 × 30 × 30 cm) constructed with polyvinyl chloride, plywood and acrylic as previously described (Wu et al., 2017). The task included training and retention over two consecutive days. On day 1 of training session, the animals explored two identical objects (black plastic cubes, 5 × 5 × 5 cm) for 5 min. On day 2 of recognition session, one of the objects was replaced by a gray plastic square pyramid with a new shape and color (5 × 5 × 7 cm), and the animals were again acclimated to the area for 5 min. The field was decontaminated with 10% ethanol solution and dry cloth between the tests. The animals explored the test area by sniffing or touching the objects with their nose and/or forepaws at a distance of less than 2 cm. Sitting or turning around the objects was not considered as exploratory behavior. The exploratory behavior was manually evaluated using a video camera by an observer blinded to the test conditions. Total exploration time referred to the amount of time devoted to location of the two objects. The cognitive function was measured using a recognition index, which was the exploration time involving either of the two objects (training session) or the novel object (retention session) compared with the total exploration time.

### Morris water maze tests

Spatial memory was tested by the Morris water maze tests as previously described (Huang et al., 2016). The water maze consisted of a circular pool (110 cm in diameter) filled with water at 23 ± 2°C and a platform. The platform was always positioned in the middle of the northwest quadrant except for on the last day. Swimming was recorded by a video camera linked to a computer-based image system. Learning was evaluated in four consecutive days. Each mouse was trained to locate the platform during four trials daily. The time required to enter the hidden platform was measured. On the last day, a probe trial was conducted by removing the platform and training the mice to swim for 90 s to locate it. Swimming time in the four quadrants of the pool was calculated. Preference for a previous quadrant occupied by the platform indicated spatial memory.

### Brain tissue collection

One day after the Morris water maze tests, animals were deeply anesthetized and transcardially perfused with ice-cold saline. Brains were dissected quickly. Proteins in the hippocampus and the cortex were extracted and stored at −80°C before use for measurements of enzyme linked immunosorbent assay (ELISA, 3 mice per group) and Western blotting assay (4 mice per group). Brain tissues (3 mice per group) were stored at −80°C before use for immunohistochemical (IHC) staining.

### ELISA assay

The concentrations of interleukin-6 (IL-6) and tumor necrosis factor-α (TNF-α) in the hippocampal and cortical regions of mice were determined from freshly prepared brain tissues. Brain samples were homogenized in 0.1 M PBS. After sonication, brain extracts were centrifuged to 2,000 g for 15 min at 4°C. The concentrations of IL-6 and TNF-α were quantified by ELISA kits (Excell bio, Shanghai, China) according to the manufacturer's protocol. The absorbance of the plates was read by a microplate reader.

### Western blotting assay

Western blotting assay was conducted as described previously (Yu et al., 2017a). Briefly, brain tissue from hippocampal region was extracted at for 1 min using a lysis buffer, and centrifuged at 16,000 g for 10 min. The protein levels in the supernatant were estimated by Bradford assay, followed by SDS-PAGE of tissue samples (40 μg), and transfer to polyvinylidene fluoride membrane. The membranes were blocked with 5 % non-fat milk in TBST for 2 h, and incubated overnight at with primary antibodies against total tau (Santa Cruz), pSer9-GSK3β, GSK3β, synapsin-1, PSD-95 and β-actin (Cell Signaling Technology, Beverly, MA, USA). After washing the samples three times with TBST, the membranes were incubated with a secondary antibody. Blots were developed using enhanced chemiluminescence as instructed by the manufacturer (Amersham Bioscience, Aylesbury, UK). All data were representative of three independent experiments. Data were expressed as ratios of optical density (OD) compared with controls for statistical analyses.

### IHC staining

IHC staining was performed according to a previously reported protocol with minor modifications (Yu et al., 2017a). Briefly, after the behavioral tests, brains were dissected and incubated with 4% paraformaldehyde for 1 day. The brain specimens were dehydrated, embedded in paraffin and cut into 4-μm-thick sections. The hippocampal sections were dewaxed and rehydrated. Treatment with 3% H_2_O_2_ for 10 min was carried out to inhibit the cellular peroxidase activity. The sections were incubated with primary antibodies against pSer396-Tau (pTau, Abcam, Eugene, OR, USA) and glial fibrillary acidic protein (GFAP, Abcam) at 4°C overnight. The specimens were rinsed and incubated with secondary antibodies at 37°C for 30 min. The sections were labeled with DAB and colorimetrically analyzed. Image Pro 6.0 (Media Cybernetics Inc., MD, USA) was used to analyze mean optical density (OD) of areas. Briefly, the digital images were used for developing semi-automated analysis. A color de-convolution technique was first used. The positive area and OD were determined by measuring three randomly selected microscopic fields for each slide. The IHC index was defined as mean OD = positive area × OD/total area (Gu et al., 2011; Chatterjee et al., 2013).

### Data analysis and statistics

Data were expressed as means ± SD. Statistical significance was determined by one-way ANOVA and Tukey's test for post hoc multiple comparison, with the exception of mean escape latency, which was analyzed using two-way repeated-measures ANOVA followed by LSD post hoc test. A *p* < 0.05 was considered as statistically significant.

## Results

### 7Bio is one of the most potent indirubin derivatives to inhibit Aβ oligomer-induced neuronal death

**Figure 1:**
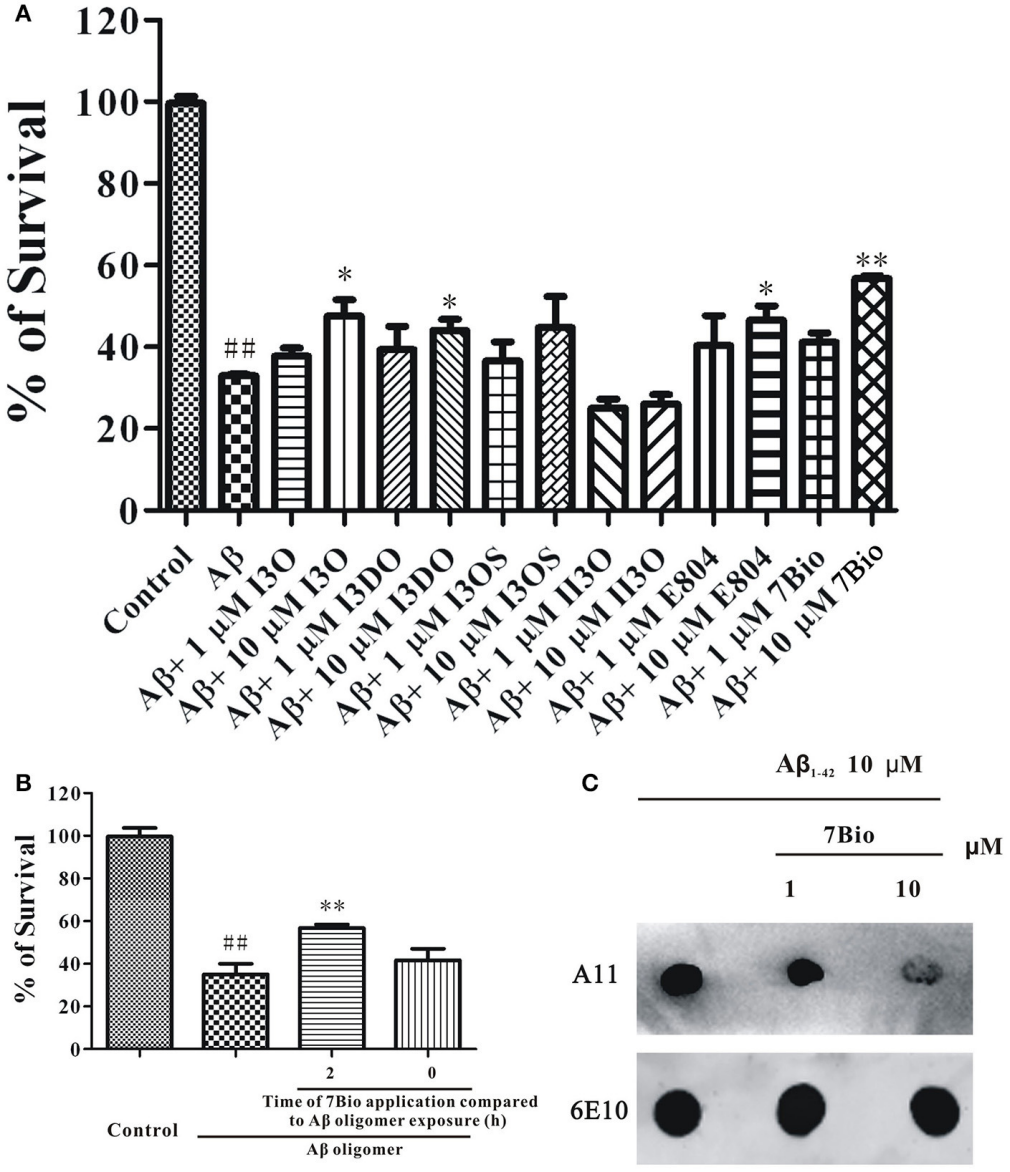
7Bio is one of the most potent chemical to inhibit Aβ oligomer-induced neuronal death in SH-SY5Y cells. **(A)** SH-SY5Y cells were treated with indirubin-3-oxime (I3O), indirubin-3-(2,3-dihydroxypropyl)oximether (I3DO), indirubin-3-monoxime-5-sulphonic acid (I3OS), 5-iodo-indirubin-3-monoxime (II3O), indirubin derivative E804 (E804), or 7Bio at various concentrations as indicated. After 2 h, 1.5 μM Aβ oligomers were added. The MTT assay was used to measure cell viability at 24 h after the addition of Aβ oligomers. **(B)** 7Bio prevents but does not rescue Aβ oligomer-induced neurotoxicity in SH-SY5Y cells. SH-SY5Y cells were exposed to 1.5 μM Aβ oligomer with 2 h pre-treatment (2 h) or co-treatment (0 h) of 10 μM 7Bio. The MTT assay was applied at 24 h after adding Aβ oligomer. Data, expressed as percentage of the control, were the mean ± SEM of three separate experiments; ^##^*p* < 0.01 vs. the control group, ^*^p < 0.05 and ^**^p < 0.01 vs. Aβ oligomer group (ANOVA and Tukey's test). **(C)** 7Bio reduces Aβ oligomer formation. Aβ peptide was co-incubated with 7Bio at the indicated doses for 2 days. Solution was centrifuged, and the supernatants were examined via dot blotting analysis with A11, an anti-oligomer antibody, and 6E10, an anti-Aβ antibody, respectively.

Previously, we have evaluated the neuroprotective effects of many indirubin derivatives, and found that 7Bio is one of the most potent chemical to inhibit Aβ oligomer-induced neuronal death in SH-SY5Y cells (Figure 1A). To explore whether 7Bio reverses or prevents neurotoxicity induced by Aβ_1−42_ oligomer, we evaluated cell viability by the MTT assay. The time-dependent neuroprotective profile of 7Bio against neurotoxicity caused by Aβ_1−42_ oligomer was determined. Pre-treatment, but not co-treatment of 7Bio decreased Aβ_1−42_ oligomer-induced cell death, suggesting that 7Bio prevented, but did not rescue Aβ_1−42_ oligomer-induced cell death in SH-SY5Y cells (Figure 1B).

To further examine if 7Bio could alter toxic Aβ oligomer formation, we used dot blotting assay *in vitro*. In a control test, Aβ_1−42_ peptide formed Aβ_1−42_ oligomer after 2 days of incubation under stirring. However, co-incubation of 7Bio largely reduced the amounts of Aβ_1−42_ oligomer compared to the control condition, suggesting that 7Bio might inhibit toxic Aβ oligomer formation (Figure 1C).

### 7Bio does not significantly change body weight and locomotor activity of mice

**Figure 2:**
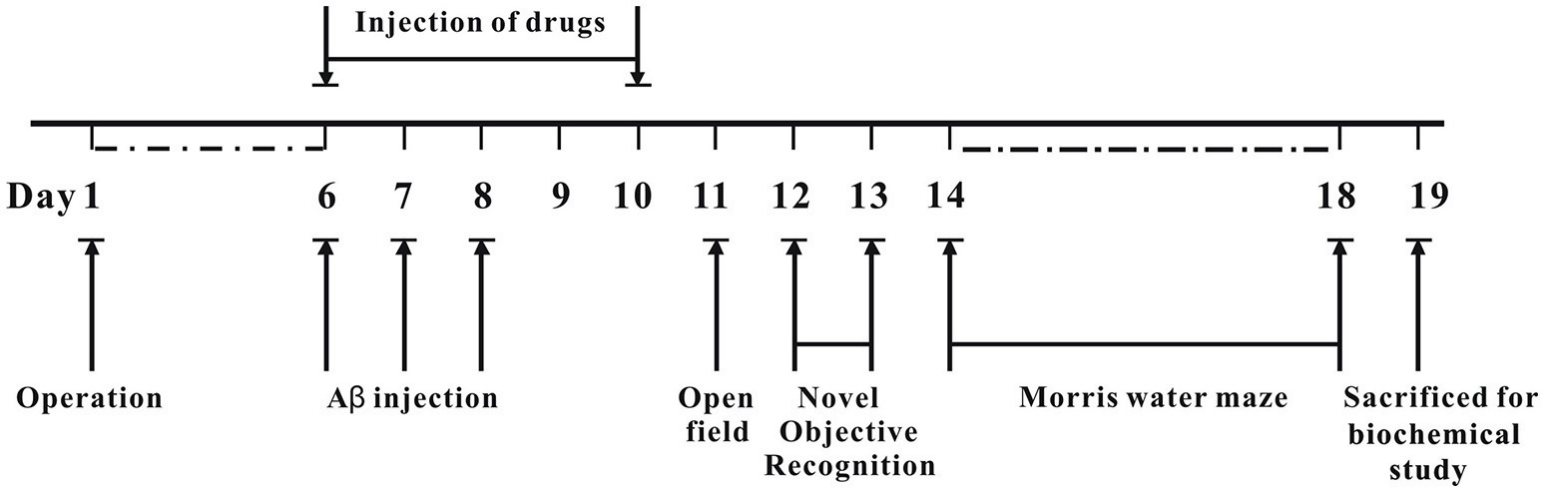
Experimental design and schedule of animal tests.

The whole process of animal experiments is shown in Figure 2. Briefly, operation was performed to implant cannulae into bilateral ventricle regions of mice. The mice were allowed to recover for 6 days. On day 6 post-operation, Aβ_1−42_ oligomer was administrated into bilateral ventricle regions for 3 days, various concentrations of 7Bio with Aβ_1−42_ oligomer, or saline were administrated into bilateral ventricle regions for 5 days. The body weight of mice was evaluated every day during experiments. The effects of 7Bio and Aβ_1−42_ oligomer on motor function were examined by the open field test on day 11 post-operation. The effects of 7Bio on Aβ oligomer-induced impairments of cognition was tested by the NOR test and the Morris water maze test, on days 12–13 and days 14–18 post-operation, respectively. At day 19 post-operation, mice were sacrificed for biochemical study.

**Figure 3:**
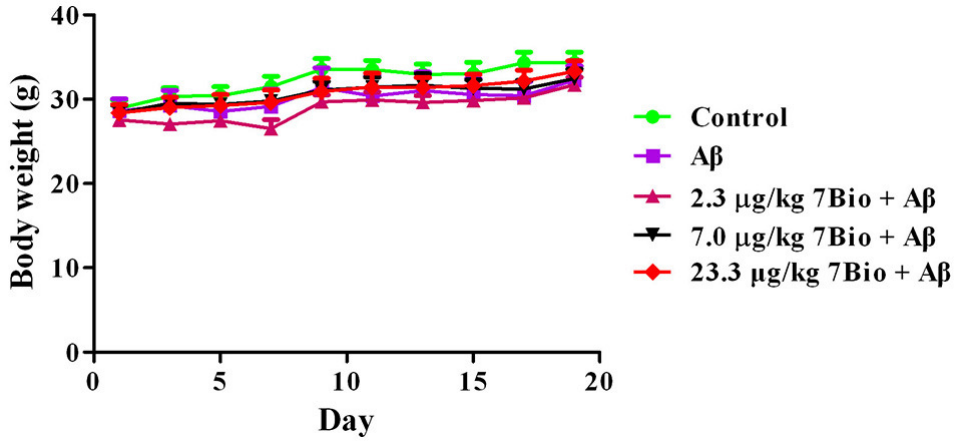
Treatments do not significantly alter the body weight of mice during the experiments. Data are expressed as the mean ± SD (*n* = 10).

Two-way repeated-measures ANOVA of bodyweight revealed that there are significant changes for time effect [two-way ANOVA *F*_(9, 450)_ = 13.472, *p* < 0.01] and treatment effect [two-way ANOVA *F*_(4, 450)_ = 6.511, *p* < 0.01], but not for treatment × time interaction [*F*_(36, 450)_ = 0.198, *p* > 0.05; Figure 3]. Further analysis suggested that the bodyweight of mice among various groups were not significantly changed during the experiments (Figure 3).

**Figure 4:**
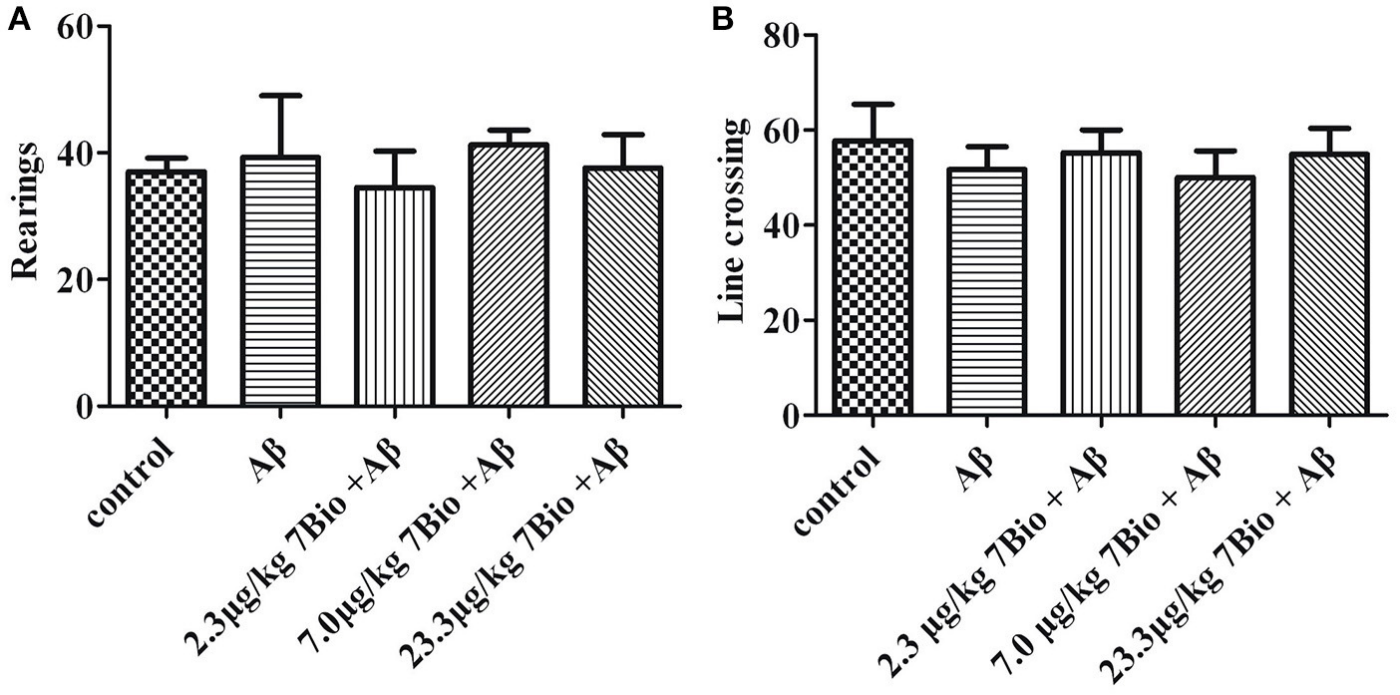
Treatments do not significantly alter motor function of mice in the open field tests. The number of rearings and line crossings were recorded and calculated as shown in **(A)** and **(B)**, respectively. Data are expressed as the mean ± SD (*n* = 10).

To examine whether surgery, Aβ_1−42_ oligomer or 7Bio affect motor function of animals, locomotor activity was first evaluated by the open field test at day 11 post-surgery. As shown in Figure 4, treatments did not significantly changed the number of rearings or line crossing in the open field test, suggesting that surgery, Aβ_1−42_ oligomer and 7Bio did not affect motor function of mice in our study [for rearing, one way ANOVA, *F*_(4, 55)_ = 0.427, *p* > 0.05, Figure 4A; for line crossing, *F*_(4, 55)_ = 0.897, *p* > 0.05, Figure 4B].

### 7Bio significantly attenuates Aβ oligomer-induced impairment of recognition in mice

**Figure 5:**
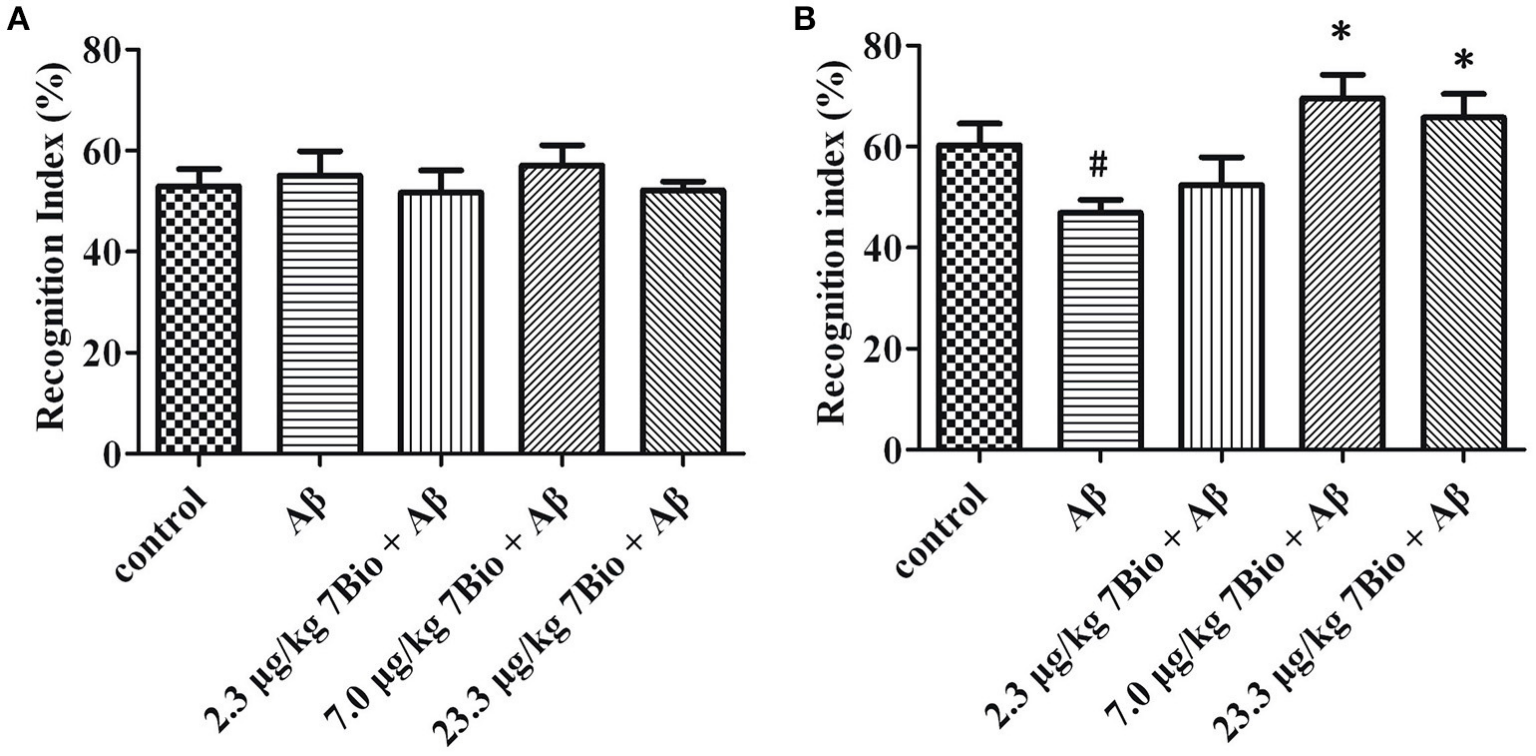
7Bio attenuates Aβ oligomer-induced cognitive impairments in mice**. (A)** In the 1st day of NOR, the recognition index was not significantly altered among various groups as indicated. **(B)** In the 2nd day of NOR, 7Bio significantly attenuated the reduction of recognition index induced by Aβ oligomer in mice. Data represent the mean ± SD (*n* = 10); ^#^*p* < 0.05 vs. control group, ^*^p < 0.05 vs. Aβ group (one-way ANOVA and Tukey's test).

The NOR test was used to examine the recognition potential of mice. On day 12 post-surgery, the training session of the NOR test, the recognition index was not significantly changed [one-way ANOVA, *F*_(5, 54)_ = 0.222, *p* > 0.05, Figure 5A]. On day 13 post-surgery, the recognition session of the NOR test, the recognition index was significantly altered among various groups [one-way ANOVA, *F*_(5, 54)_ = 4.339, *p* < 0.01, Figure 5B]. Furthermore, the recognition index in the Aβ_1−42_ oligomer group was significantly lower than that in the control group [one-way ANOVA, Tukey's test, *p* < 0.05, Figure 5B]; the recognition index in 7.0 μg/kg 7Bio + Aβ_1−42_ oligomer and 23.3 μg/kg 7Bio + Aβ_1−42_ oligomer groups were significantly higher than that in the Aβ_1−42_ oligomer group (one-way ANOVA, Tukey's test, *p* < 0.05, Figure 5B). These results suggested that 7Bio could prevent Aβ_1−42_ oligomer-induced impairments of recognition in mice.

### 7Bio significantly attenuates Aβ oligomer-induced impairment of spatial learning and memory in mice

**Figure 6:**
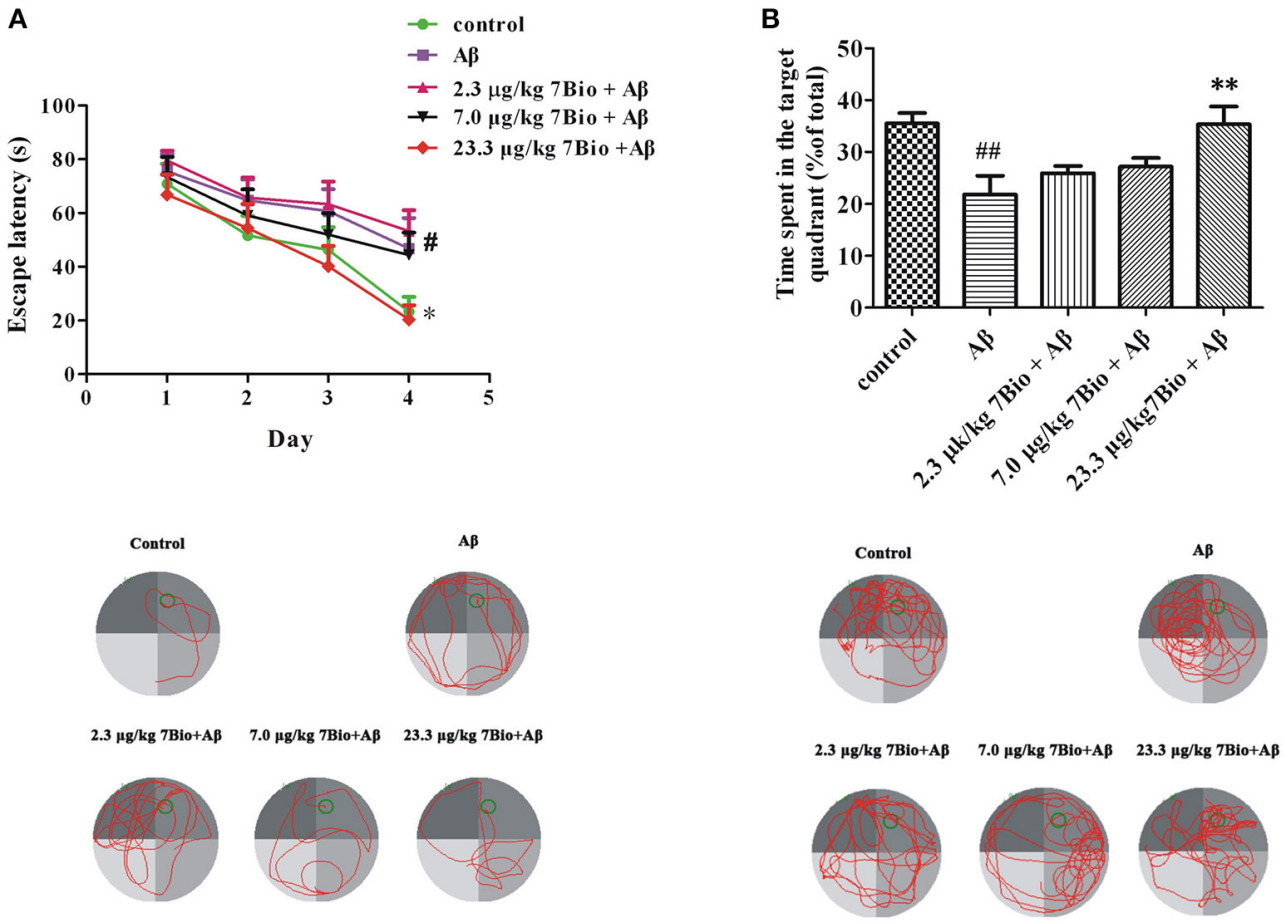
7Bio attenuates Aβ oligomer-induced impairments of spatial learning and memory in mice. **(A)** During the 4 days of training session, 7Bio at 23.3 μg/kg significantly reduced the escape latency of Aβ oligomer-treated mice to find the hidden platform in the Morris water maze test. Lower part: representative tracking paths of various groups as indicated in the last day of training session. **(B)** In the probe trial, 7Bio at 23.3 μg/kg significantly attenuated the decreased duration of Aβ oligomer-treated mice in the target quadrant in the Morris water maze test. Lower part: representative tracking paths of various groups as indicated in the probe trial. Data represent the mean ± SD (*n* = 10); ^#^*p* < 0.05 and ^##^*p* < 0.01 vs. the control group; ^*^p < 0.05 and ^**^p < 0.01 vs. the Aβ group (one-way ANOVA and Tukey's test).

The spatial learning and memory was further evaluated by Morris water maze test. At day 14–17 post-surgery, the training session of Morris water maze test, two-way repeated-measures ANOVA revealed significant changes in time effect [two-way ANOVA, *F*_(3, 180)_ = 17.95, *p* < 0.01, Figure 6A] and treatment effect [two-way ANOVA, *F*_(4, 180)_ = 5.03, *p* < 0.01, Figure 6A], but not in treatment × time interaction [*F*_(12, 180)_ = 0.413, *p* > 0.05; Figure 6A]. Further analysis indicated that on the last day of the training session, mice in the Aβ_1−42_ oligomer group spend a significantly longer time to find the platform when compared with mice in the control group (*p* < 0.05, Figure 6A). Moreover, mice in 23.3 μg/kg 7Bio + Aβ_1−42_ oligomer group spend a significantly shorter time to reach the platform when compared with mice in the Aβ_1−42_ oligomer group (*p* < 0.05, Figure 6A). These results suggested that 7Bio could attenuate Aβ_1−42_ oligomer-induced impairments of spatial learning in animals.

At day 18 post-surgery, the probe trial of Morris water maze test, the time spent in the target quadrant was recorded. The time spend in the target quadrant was significantly different among various groups [one-way ANOVA, *F*_(5, 54)_ = 5.653, *p* < 0.01, Figure 6B]. Interestingly, mice in the Aβ_1−42_ oligomer group spend significantly shorter time in the target quadrant when compared with mice in the control group (*p* < 0.01, Figure 6B). Mice in 23.3 μg/kg 7Bio + Aβ_1−42_ oligomer group spend significantly longer time in the target quadrant when compared with mice in the Aβ_1−42_ oligomer group (*p* < 0.01, Figure 6B). These results suggested that 7Bio could also prevent Aβ_1−42_ oligomer-induced impairments of spatial memory in animals.

### 7Bio decreases Aβ oligomer-induced increase of TNF-α and IL-6 production in the brain of mice

**Figure 7:**
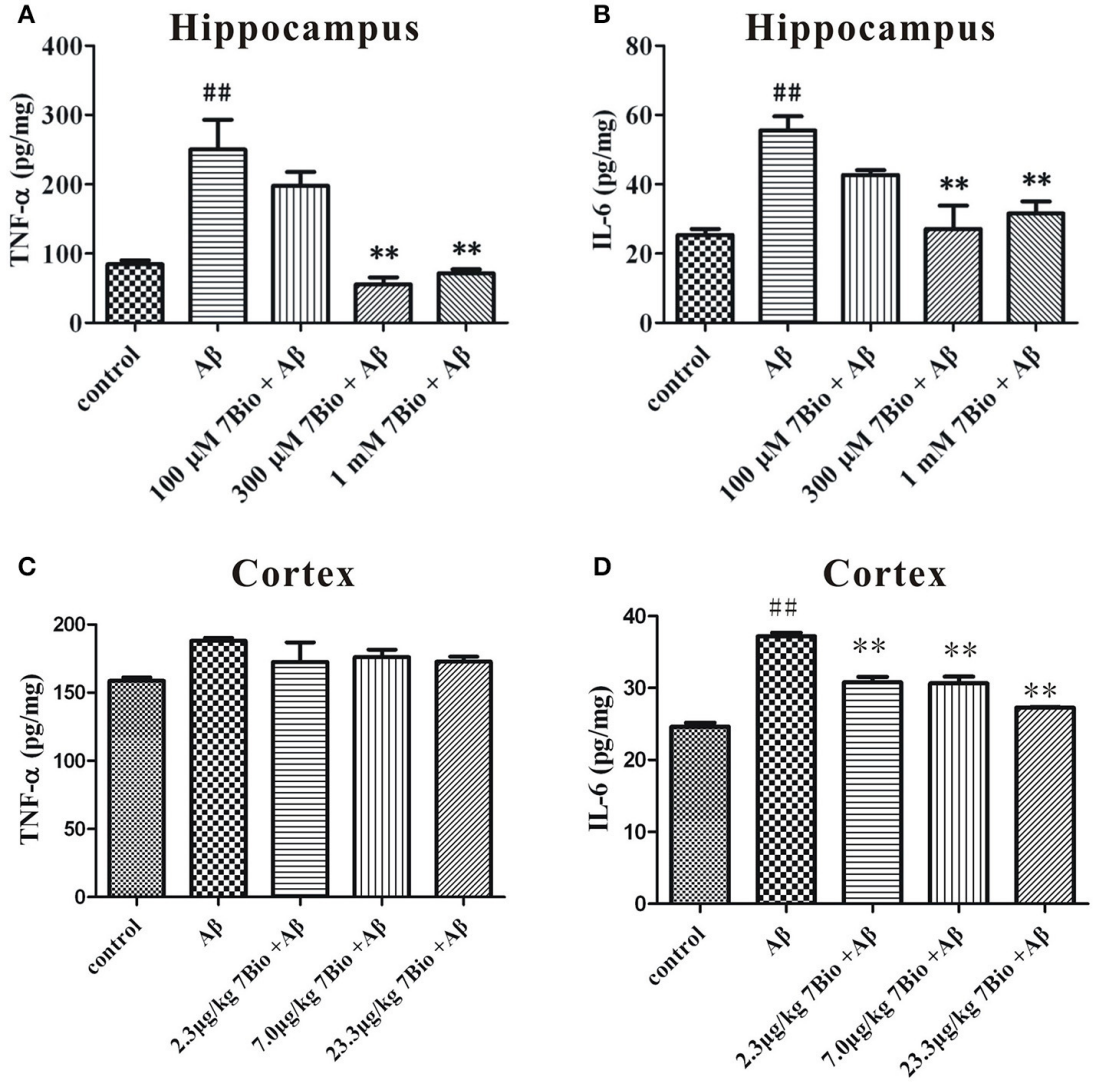
7Bio attenuates Aβ oligomer-induced increase of TNF-α and IL-6 in mice. At day 19 post-surgery, mice were sacrificed. The levels of TNF-α **(A** for hippocampus and **C** for cortex) and IL-6 **(B** for hippocampus and **D** for cortex) were evaluated by ELISA assay in the hippocampal and cortical extraction of mice, respectively. Data represent the mean ± SD (*n* = 3); ^##^*p* < 0.01 vs. control group, ^**^p < 0.01 vs. Aβ group (one-way ANOVA and Tukey's test).

We further evaluated the production of TNF-α and IL-6 by using ELISA in the hippocampal and cortical region of mice. On day 19 post-surgery, mice were sacrificed and the hippocampus and the cortex were dissected and extracted. The levels of TNF-α and IL-6 were significantly higher in the hippocampal region of mice in the Aβ_1−42_ oligomer group than those in the control group (*p* < 0.01, Figures 7A,B). Moreover, 7Bio at 7.0 and 23.3 μg/kg significantly decreased the production of TNF-α and IL-6 in the hippocampal region of mice (*p* < 0.01, Figures 7A,B). For the cortical region, the level of IL-6 but not TNF-α were significantly higher the Aβ_1−42_ oligomer group than those in the control group (*p* < 0.01, Figures 7C,D). Moreover, 7Bio significantly reduced the production of IL-6 in the cortical region of mice (*p* < 0.01, Figure 7D).

### 7Bio increases Aβ oligomer-induced decreased expression of synapsin-1 and PSD-95 in the hippocampal region of mice

**Figure 8:**
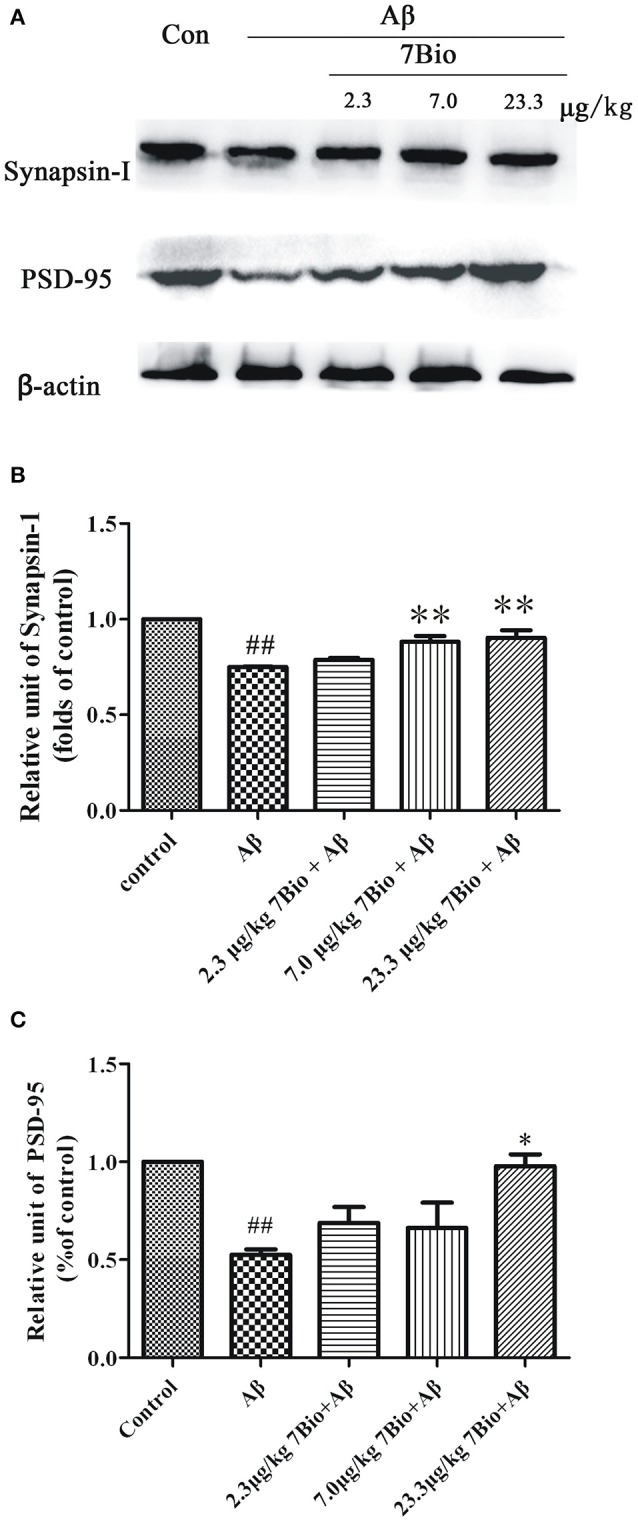
7Bio attenuates Aβ oligomer-induced decrease expression of synapsin-1 and PSD-95 in mice. At day 19 post-surgery, mice were sacrificed. **(A)** The expressions of synapsin-1, PSD-95 and β-actin were evaluated by Western blotting analysis. The quantitative analysis of levels of synapsin-1 and PSD-95 were shown in **(B)** and **(C)**, respectively. Data represent the mean ± SD (*n* = 4); ^##^*p* < 0.01 vs. control group, ^*^p < 0.05 and ^**^p < 0.01 vs. Aβ group (one-way ANOVA and Tukey's test).

Synapsin-1 and PSD-95 are pre- and post- synaptic proteins, respectively. In this study, we used Western blotting assay to analyze the expression of these two proteins in the hippocampal region of mice. The expression of synapsin-1 and PSD-95 were significantly lower in the hippocampal region of mice in the Aβ_1−42_ oligomer group than those in the control group (*p* < 0.01, Figure 8). Furthermore, 7Bio at 7.0 and 23.3 μg/kg significantly increased the expression of synapsin-1 in the hippocampal region of mice when compared with the Aβ_1−42_ oligomer group (*p* < 0.01, Figure 8). 7Bio at 23.3 μg/kg significantly increased the expression of PSD-95 when compared with the Aβ_1−42_ oligomer group (*p* < 0.05, Figure 8). These results suggested that 7Bio increases Aβ oligomer-induced decreased expression of synapsin-1 and PSD-95 in the hippocampal region of mice.

### 7Bio attenuates Aβ oligomer-induced increase expression of pTau in the hippocampal region of mice

**Figure 9:**
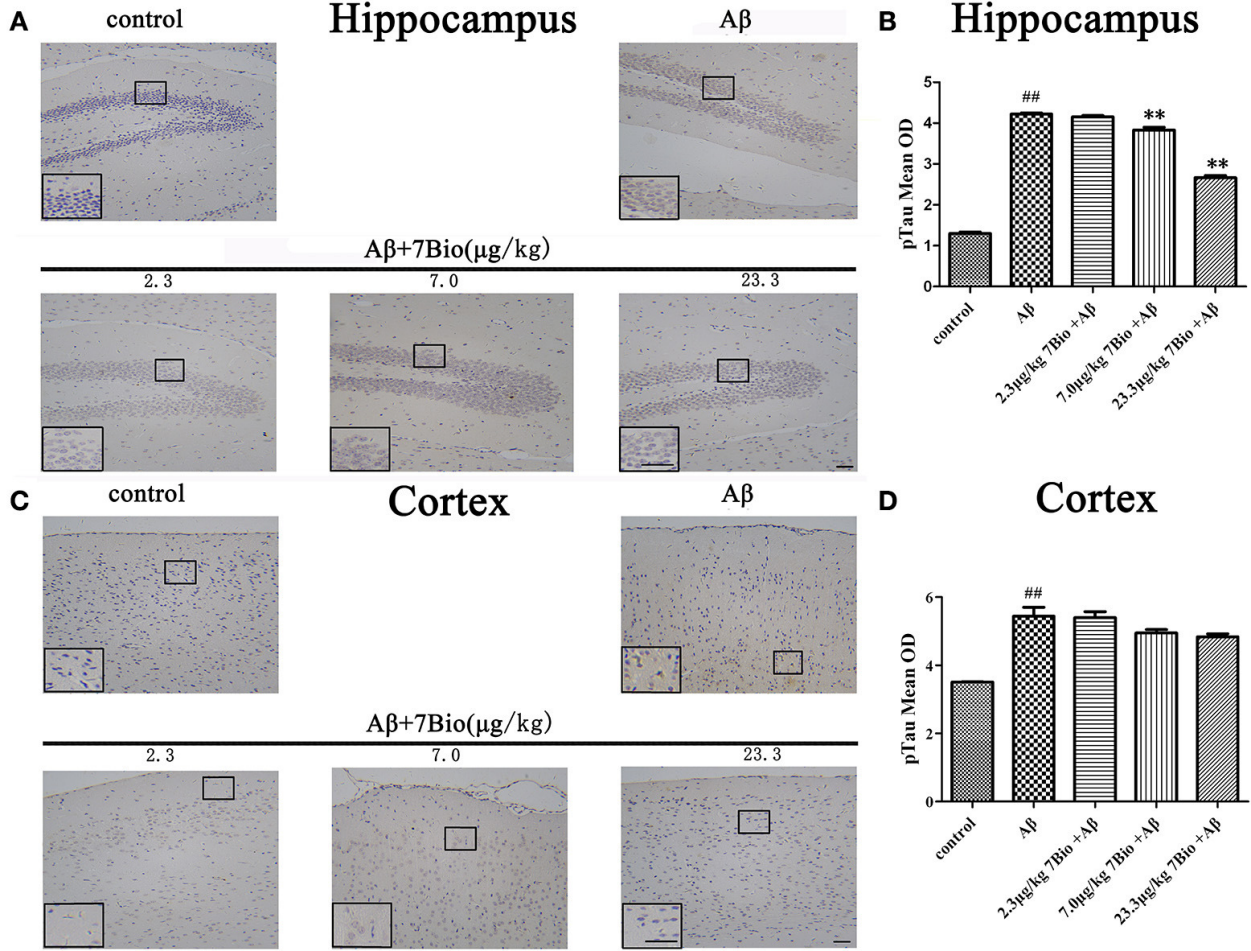
7Bio attenuates Aβ oligomer-induced increased of pTau in the hippocampal region of mice. **(A)** Representative figures of IHC staining of pTau in the hippocampal region in various groups as indicated. **(B)** Quantitative results showed that 7Bio decreased mean OD of pTau in the hippocampal region of mice. **(C)** Representative figures of IHC staining of pTau in the cortical region in various groups as indicated. **(D)** Quantitative results showed that Aβ oligomer-induced increased mean OD of pTau in the cortical region of mice. Data represent the mean ± SD (*n* = 3); ^##^*p* < 0.01 vs. control group, ^**^p < 0.01 vs. Aβ group (one-way ANOVA and Tukey's test). Scale bar: 30 μm.

Hyper-phosphorylation of tau protein is a hallmark of AD. Therefore, we further used IHC to evaluate the expression of pTau in the hippocampal and cortical region of mice. Injection of Aβ_1−42_ oligomer significantly increased the mean OD of pTau in the hippocampal and cortical region when compared to the control group (*p* < 0.01, Figure 9). Moreover, 7Bio at 7.0 and 23.3 μg/kg significantly prevented the mean OD of pTau in the hippocampal region when compared with the Aβ_1−42_ oligomer group (*p* < 0.01, Figures 9A,B).

### 7Bio attenuates Aβ oligomer-induced astrogliosis in the brain of mice

**Figure 10:**
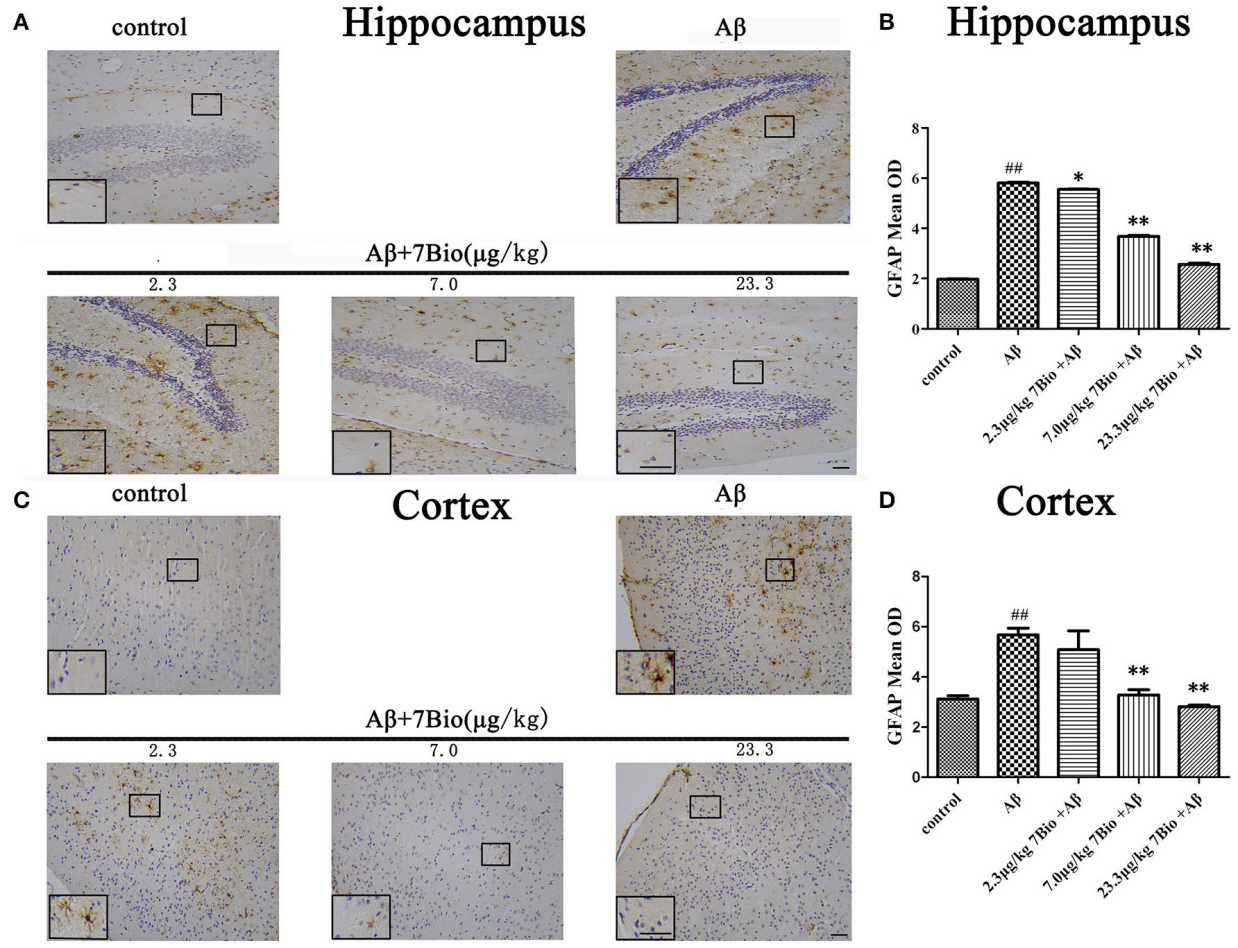
7Bio attenuates Aβ oligomer-induced increased of GFAP in the hippocampal and cortical region of mice. **(A)** Representative figures of IHC staining of GFAP in the hippocampal region in various groups as indicated. **(B)** Quantitative results showed that 7Bio decreased mean OD of GFAP in the hippocampal region of mice. **(C)** Representative figures of IHC staining of GFAP in the hippocampal region in various groups as indicated. **(D)** Quantitative results showed that 7Bio decreased mean OD of GFAP in the cortical region of mice. Data represent the mean ± SD (*n* = 3); ^##^*p* < 0.01 vs. control group, ^*^p < 0.05 and ^**^p < 0.01 vs. Aβ group (one-way ANOVA and Tukey's test). Scale bar: 30 μm.

GFAP is a biomarker of astrogliosis. To investigate whether 7Bio affected Aβ_1−42_ oligomer-induced astrogliosis, IHC staining of GFAP was performed in our study. Injection of Aβ_1−42_ oligomer significantly increased the mean OD of GFAP in the hippocampal and cortical region when compared to the control group (*p* < 0.01, Figure 10). Moreover, 7Bio at 2.3, 7.0, and 23.3 μg/kg significantly prevented the mean OD of GFAP in the hippocampal region when compared with the Aβ_1−42_ oligomer group (*p* < 0.05, Figures 10A,B). 7Bio at 7.0 and 23.3 μg/kg significantly decreased the mean OD of GFAP in the cortical region when compared with the Aβ_1−42_ oligomer group (*p* < 0.01, Figures 10C,D).

### 7Bio reduces Aβ oligomer-induced activation of microglia in the brain of mice

**Figure 11:**
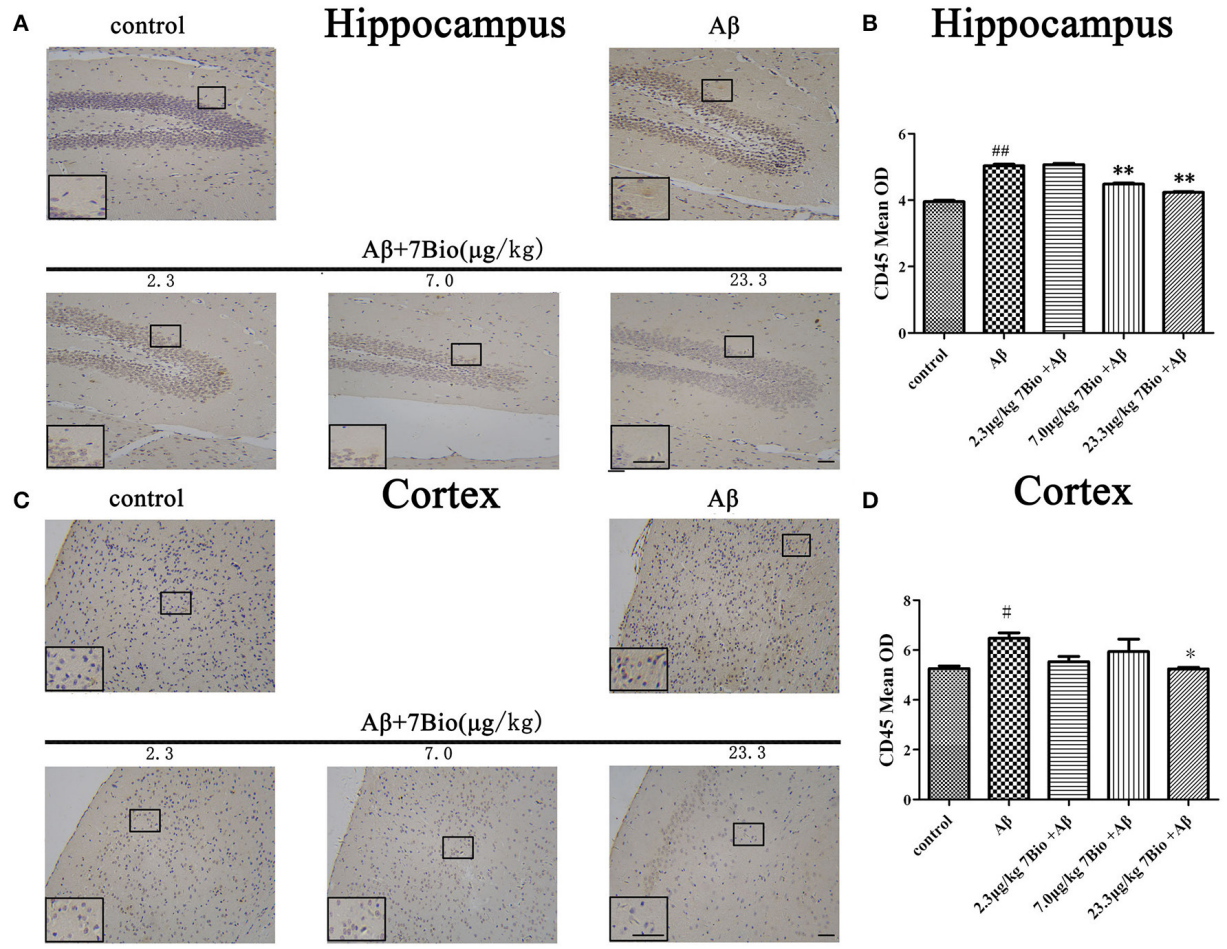
7Bio attenuates Aβ oligomer-induced increased of CD45 in the hippocampal and cortical region of mice. **(A)** Representative figures of IHC staining of CD45 in the hippocampal region in various groups as indicated. **(B)** Quantitative results showed that 7Bio decreased mean OD of CD45 in the hippocampal region of mice. **(C)** Representative figures of IHC staining of CD45 in the hippocampal region in various groups as indicated. **(D)** Quantitative results showed that 7Bio decreased mean OD of CD45 in the cortical region of mice. Data represent the mean ± SD (*n* = 3); ^#^*p* < 0.05 and ^##^*p* < 0.01 vs. control group, ^*^p < 0.05 and ^**^p < 0.01 vs. Aβ group (one-way ANOVA and Tukey's test). Scale bar: 30 μm.

CD45 is a biomarker of microglia in the brain. To investigate whether 7Bio affected Aβ_1−42_ oligomer-induced activation of microglia, IHC staining of CD45 was performed in our study. Injection of Aβ_1−42_ oligomer significantly increased the mean OD of CD45 in the hippocampal and cortical region when compared to the control group (*p* < 0.05, Figure 11). Moreover, 7Bio at 7.0 and 23.3 μg/kg significantly prevented the mean OD of CD45 in the hippocampal region when compared with the Aβ_1−42_ oligomer group (*p* < 0.01, Figures 11A,B). 7Bio at 23.3 μg/kg significantly decreased the mean OD of CD45 in the cortical region when compared with the Aβ_1−42_ oligomer group (*p* < 0.05, Figures 11C,D).

### 7Bio prevents Aβ oligomer-induced decreased expression of pSer9-GSK3β in the hippocampal region of mice

**Figure 12:**
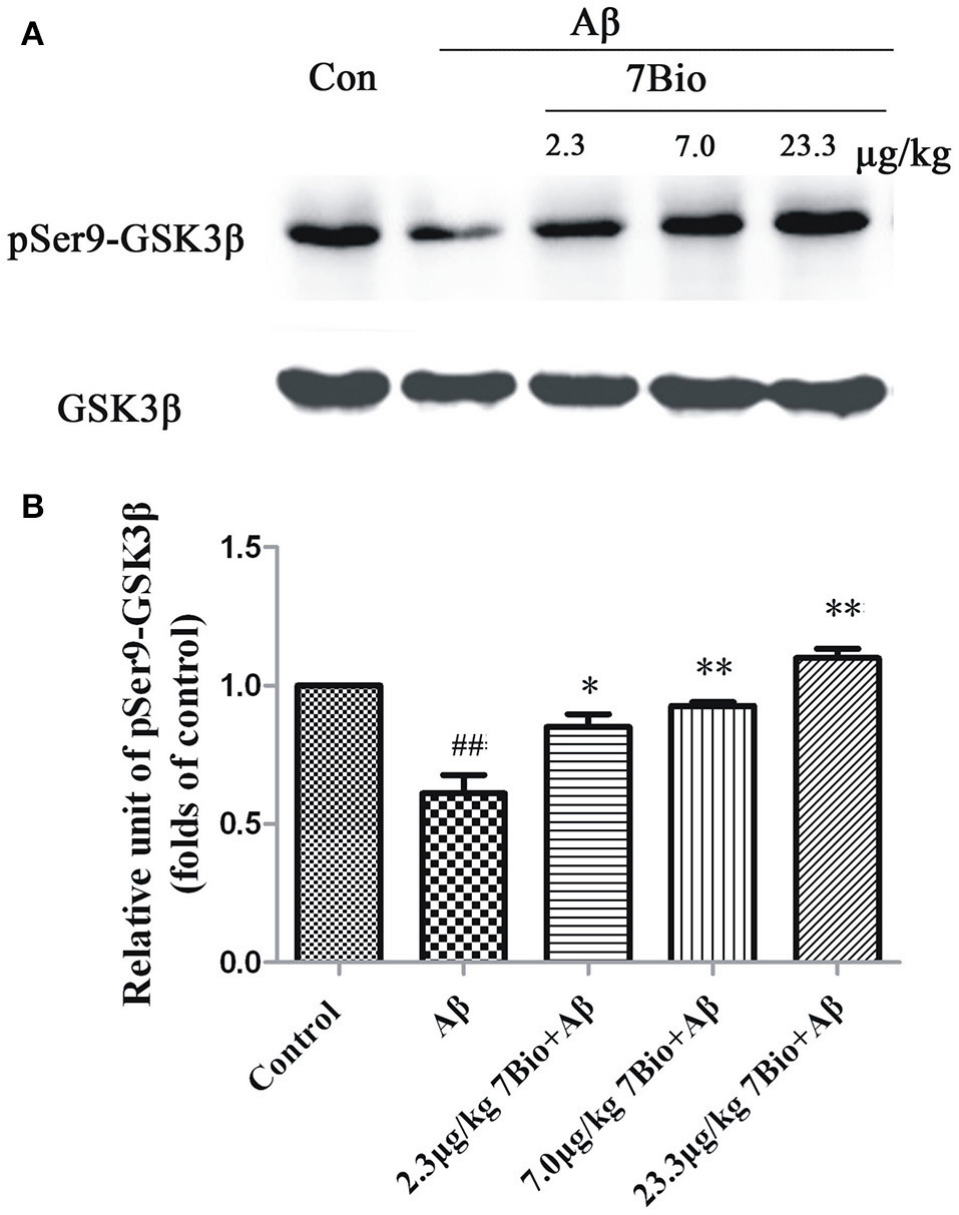
7Bio attenuates Aβ oligomer-induced decrease expression of pSer9-GSK3β in the hippocampal region of mice. At day 19 post-surgery, mice were sacrificed. **(A)** The expressions of pSer9-GSK3β and GSK3β in the hippocampus were evaluated by Western blotting analysis. The quantitative analysis of levels of pSer9-GSK3β was shown in **(B)**. Data represent the mean ± SD (*n* = 4); ^##^*p* < 0.01 vs. control group, ^*^p < 0.05 and ^**^p < 0.01 vs. Aβ group (one-way ANOVA and Tukey's test).

To explore whether 7Bio could affect the activity of GSK3β *in vivo*, the expression of pSer9-GSK3β in the hippocampal region was evaluated by Western blotting assay. 7Bio significantly increased the expression of pSer9-GSK3β in the hippocampal region of mice when compared with the Aβ_1−42_ oligomer group (*p* < 0.05, Figure 12), suggesting that 7Bio might inhibit the activity of GSK3β in Aβ oligomer-treated mice.

### 7Bio did not significantly alter Aβ oligomer-induced decreased expression of Tau protein in the hippocampal region of mice

**Figure 13:**
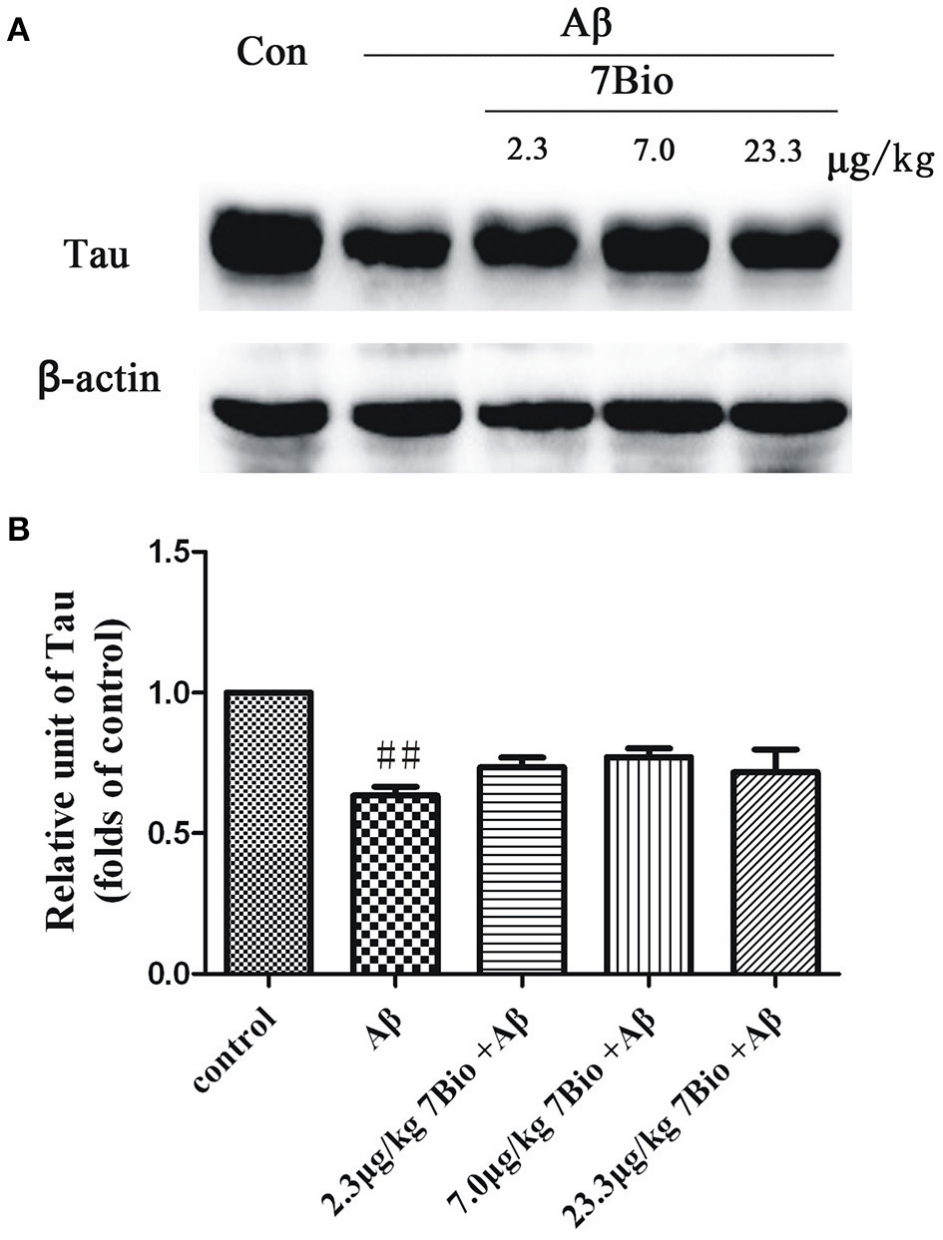
7Bio did not significantly prevent Aβ oligomer-induced decrease expression of tau protein in the hippocampal region of mice. At day 19 post-surgery, mice were sacrificed. **(A)** The expressions of tau protein and β-actin in the hippocampus were evaluated by Western blotting analysis. The quantitative analysis of levels of tau protein was shown in **(B)**. Data represent the mean ± SD (*n* = 4); ^##^*p* < 0.01 vs. control group (one-way ANOVA and Tukey's test).

To explore whether 7Bio could affect the expression of tau protein, the Western blotting assay was used. The expression of tau protein was significantly lower in the hippocampal region of mice in the Aβ_1−42_ oligomer group than those in the control group (*p* < 0.01, Figure 13). However, 7Bio did not significantly alter the expression of tau protein in the hippocampal region of mice when compared with the Aβ_1−42_ oligomer group (Figure 13), suggesting that 7Bio might not substantially affect tau expression in our system.

## Discussion

In this study, we have reported, for the first time, that 7Bio effectively attenuated Aβ oligomer-induced cognitive impairments. We have also found that 7Bio significantly prevented the increase of neuroinflammation, the reduction of synaptic proteins, the hyper-phosphorylation of tau protein, and the activation of astrocytes and microglia, possibly via inhibiting GSK3β in the hippocampal region of mice.

AD is characterized by the impairments of cognitive functions. Aβ oligomer is considered as the main neurotoxin of AD (Viola and Klein, 2015; Selkoe and Hardy, 2016). Previous studies have shown that the injection of Aβ oligomer into the brain could induce cognitive impairments in rodents (Pi et al., 2012; Chang et al., 2015). Therefore, Aβ oligomer injection is traditionally used to establish animal models of AD. In our study, Aβ oligomer was administrated into the bilateral ventricle regions of mice. Three days injection of Aβ oligomer led to the significant impairments of spatial cognition and recognition without affecting bodyweight and motor functions, which was consistence with previous studies (Viola and Klein, 2015; Selkoe and Hardy, 2016).

Accumulating evidence suggested that the activities of CDK5 and GSK3β are elevated in AD (Leclerc et al., 2001; Tell et al., 2012). Many CDK5 and GSK3β inhibitors are reported to produce anti-AD neuroprotective effects (Leclerc et al., 2001; Tell et al., 2012). Thus, 7Bio, a moderate inhibitor of CDK5 and GSK3β, might also exhibit anti-AD property. Previously, we have found many indirubin derivatives could prevent neurotoxins-induced neurotoxicity (Hu et al., 2015). Moreover, 7Bio is the one of the most potent indirubin derivatives to inhibit Aβ oligomer-induced neuronal death in SH-SY5Y cells. We also found that 7Bio could prevent but not rescue Aβ oligomer-induced neuronal death, and inhibit Aβ oligomer formation *in vitro*. Therefore, we evaluated the effects of 7Bio on the prevention of cognitive impairments in our AD animal model. Our findings that 7Bio at very low concentrations, potently attenuated Aβ oligomer-induced cognitive impairments, suggested that 7Bio could enhance cognitive performance. These results are consistent with previous studies that many indirubin derivatives, such as indirubin-3-oxime could prevent cognitive impairments in APP/PS1 transgenic mice and high fat food-treated mice (Ding et al., 2010; Sharma and Taliyan, 2014).

Besides cognitive impairments, neuroinflammation is also an important feature of AD. Among various pro-inflammatory cytokines, TNF-α and IL-6 are main cytokines which contributed to neuroinflammatory process observed in AD (Morales et al., 2010). In our study, 7Bio significantly reduced the expression of pro-inflammatory cytokines TNF-α and IL-6 in Aβ oligomer-treated mice, suggesting the anti-inflammatory effects of 7Bio in the brain. These results are also consistent with previous studies that indirubin derivatives could alleviate lipopolysaccharide-induced peripheral injury via its anti-inflammatory functions (Lai et al., 2017; Qi et al., 2017). Importantly, our study is one of the first studies to report that indirubin derivatives could produce anti-inflammatory effects in the central nervous system. How could 7Bio produce anti-inflammatory effects? Previous studies have shown that many indirubin derivatives could directly regulate inflammation-associated signaling in a protein kinase-dependent manner. Many kinases, such as NF-κB and STAT3, are involved in inflammation-associated signaling (Kim and Park, 2012; Lai et al., 2017). Although not proved in this study, 7Bio might inhibit these kinases to produce anti-inflammatory effects.

Loss of synapses and synaptic functions in the cortex and hippocampus is also a prominent feature of AD. Multiple studies have previously revealed a decline in a wide range of different synaptic proteins, including synapsin-1 and PSD-95 in the process of AD (Tu et al., 2014). Synapsin-1, a pre-synaptic marker, is a phosphoprotein that specifically localized in the cytoplasm of pre-synaptic neurons (Milovanovic and De Camilli, 2017). PSD-95 is a post-synaptic marker that provided essential scaffolding for post-synaptic receptors and ion channels (Savioz et al., 2014). Our findings that 7Bio significantly attenuated Aβ oligomer-induced decreased expression of synapsin-1 and PSD-95, suggested that 7Bio might prevent synaptic toxicity in AD.

The accumulation of hyper-phosphorylated tau protein and Aβ plaques are two hallmarks of AD. Aβ oligomer could trigger the pathological phosphorylation of tau protein both *in vitro* and *in vivo*. The hyper-phosphorylation of tau protein is essentially carried out by CDK5 and GSK3β (Mazanetz and Fischer, 2007). Therefore, CDK5 and GSK3β inhibitors are expected to reduce the hyper-phosphorylation of tau protein, and regarded as lead compounds for treating AD. In our study, we found that 7Bio could reduce the expression of hyper-phosphorylated tau in the hippocampal region of Aβ oligomer-treated mice. Interestingly, many studies have shown that other indirubin derivatives could inhibit the expression of hyper-phosphorylated tau in okadaic acid-treated cultured neurons, and Aβ-treated SH-SY5Y cells (Zhang et al., 2009; Martin et al., 2011). These results provided a strong support that indirubin derivatives, as CDK5 and GSK3β inhibitors, might be developed as anti-AD lead compounds. Interestingly, there is a report showed that 6Bio, another indirubin derivative at 2.5–10 μM could decrease the expression of tau in the cultured neurons (Martin et al., 2009). We found that 7Bio did not significantly alter Aβ oligomer-induced reduction of tau expression in the hippocampal region of mice. Although 7Bio and 6Bio are very similar in chemical structure, the concentrations of chemicals used in two studies are different. Moreover, there are more influence factors, such as glial cells and blood vessels in mice than in cultured neurons. Therefore, the discrepant results of tau protein regulated by indirubin derivatives between two studies are not quite surprising.

Astrogliosis, an abnormal increase in the number of astrocytes, and activation of microglia are normally observed in neurodegenerative disorders. In our study, Aβ oligomer significantly increased the expression of GFAP and CD45, biomarkers of astrocytes and microglia, respectively, in the hippocampus of mice. These results are consistent with other studies that the activation of astrocytes and microglia is presented in AD animals (Tarantini et al., 2017). Moreover, we found that astrogliosis and the activation of microglia were decreased by 7Bio in Aβ oligomer-treated mice. Although the underlying molecular mechanisms remain unproven, we proposed that 7Bio might directly inhibit astrogliosis and microglia via inhibiting CDK5 and GSK3β, which have been reported to regulate the activation of glial cells. By using Western blotting assay, we found that 7Bio could prevent Aβ oligomer-induced decrease of pSer9-GSK3β in mice. GSK3β inhibitors were reported to protect against Aβ oligomer-induced neuroinflammation and activation of astrocytes and microglia (Maqbool et al., 2016). Therefore, we speculated that 7Bio, as a potent GSK3β inhibitor, might exert its neuroprotective effects via directly acting on GSK3β.

One limitation for the use of 7Bio in neurological diseases is the lack documentation for the toxicity, absorption and pharmacokinetics of this compound. Indirubin-3-oxime, another indirubin derivative, was reported to be detected in the brain of rats after systemically administration (Selenica et al., 2007). However, in that study, very low (17 μM) concentration of indirubin-3-oxime was found after i.p. injection of 20 mg/kg indirubin-3-oxime in rats (Selenica et al., 2007). These results also suggested that the bioavailability of indirubin derivatives is not very high. Therefore, we chose to inject drugs into bilateral ventricle regions of the brain in our study. The concentration of 7Bio in the brain could be high after intra-ventricular injection, and peripheral side effects of 7Bio could be largely limited. However, further studies of toxicity and pharmacokinetics by systemic administration are needed to develop 7Bio as a novel anti-AD lead compound.

In conclusion, we found, for the first time, that 7Bio could prevent Aβ oligomer-induced cognitive impairments, possibly via decreasing neuroinflammation, synaptic damage, tau hyper-phosphorylation, and the activation of astrocytes and microglia. Although the toxicity and pharmacokinetics of 7Bio is not clear, our findings suggested that 7Bio might be developed as a novel anti-AD lead compound after further study.

## Author contributions

Conceived and designed the experiments: WC. Performed the experiments: LC, CH, JS, MW, SY, and FZ. Analyzed the data: QW, YH, CW, and ZZ.

### Conflict of interest statement

The authors declare that the research was conducted in the absence of any commercial or financial relationships that could be construed as a potential conflict of interest.

## Abbreviations

Aβ: β-amyloid
AD: Alzheimer's disease
CDKs: cyclin-dependent kinases
CDK5: cyclin-dependent kinase-5
GFAP: glial fibrillary acidic protein
HFIP: hexafluoroisopropanol
ICR: Institute of Cancer Research
IHC: immunohistochemical
IL-6: interleukin-6
NOR: novel object recognition
OD: optical density
PD: Parkinson's disease
TNF-α: tumor necrosis factor-α
7Bio: 7-bromoindirubin-3-oxime.
